# Addressing the deficit in chronic traumatic encephalopathy research in Africa: a call for urgent attention

**DOI:** 10.1186/s41016-025-00388-5

**Published:** 2025-02-01

**Authors:** Abdulbasit Opeyemi Muili, Kehinde Alare, Oluwapelumi Samuel Solagbade, Piel Panther Kuol

**Affiliations:** 1 Department of Medicine, Ladoke Akintola University of Technology, Ogbomosho, Nigeria; 2 Department of Medicine and Surgery, Obafemi Awolowo University, Ife, Nigeria; 3 School of Medicine, Moi University, Eldoret, Kenya

## Abstract

Chronic traumatic Encephalopathy (CTE) has been linked to an increase in the mortality of contact sport athletes in the USA, most especially in the early 2000s due to limited information on its existence. The lack of understanding of its existence resulted in delayed diagnosis and inadequate treatment of the disease for affected individuals.

Africa faces a similar gap as awareness and research on CTE remain limited in the region where active participation in contact sports is rising. If no drastic action is taken to mitigate the gap, the region may face similar health consequences in the future.

Various challenges responsible for the gap can be attributed to limited infrastructure, limited funding opportunities, and sociocultural factors. To address these challenges, a multifaceted approach is necessary through increasing funding, integrating CTE education into the medical curriculum, improving infrastructure, and resolving sociocultural myths about organ donation.

Dear Editor,

Chronic traumatic encephalopathy, also called CTE, is a rare neurodegenerative disease that has been responsible for the mortality of professional contact sports players worldwide [1]. Its high mortality rate in contact sports, i.e., American football, rugby, and boxing, stems from repeated head-on collisions associated with these sports, causing trauma to the head. It poses a silent long-term threat, as its symptoms manifest months, and even several years after the injury after the initial trauma with notable symptoms including memory loss, mood swings, personality changes, and behavioral changes [2]. Despite significant research advances in North America and Europe [3, 4]. CTE remains understudied in Africa. Without intervention, Africa is at risk of delayed diagnosis and inadequate treatment which plagued the USA in the early 2000s, potentially straining healthcare systems and affecting public health outcomes in the continent [5].

In Africa, CTE is severely understudied with no large-scale epidemiological or clinical study conducted to understand its prevalence, risk factors, epidemiology, or long-term outcomes in the population. Additionally, no case series or case reports have been undertaken to show evidence of its existence in the region. This research deficit is quite alarming as contact sports like rugby and boxing are popular in countries like South Africa and Nigeria where the players are at high risk of repetitive head trauma. While American football (associated with the most cases of CTE in the world) is gaining popularity through international training programs [6], well-established sports like boxing and rugby likely pose a more immediate concern, given their longer history in the region. Despite these risks, there is no recorded data on the incidence or prevalence of CTE in Africa.

One primary challenge is the lack of diagnostic infrastructure. The post-mortem nature of CTE diagnosis [7] which requires comprehensive neuropathological examination exacerbates these challenges as there is a significant lack of advanced facilities and skilled professionals in the region. Awareness and education are also important as most health professionals and the general public in Africa are largely unfamiliar with CTE which can make its cases go unreported by affected individuals or even confused with conditions like dementia or mental health conditions by health professionals when reported.

Limited funding opportunities and sociocultural factors further impede CTE research in Africa. Clinical research in Africa is severely underfunded. The limitations in securing funds make it impossible for African researchers to conduct retrospective studies and clinical trials, leaving the research deficit unresolved. This situation is further compounded by Sociocultural factors which also negatively affect CTE research in the African region. Post-humous Brain donation has been instrumental in advancing CTE research in countries like the USA [8] but faces resistance in most African nations. Studies show that cultural and religious factors like strong moral codes on pre- or post-humous organ donation discourage post-humous brain donations which is essential for advancing neuropathological research on CTE [9–11]. For example, the Zulu group in South Africa believes that preserving deceased bodies is a way of maintaining spiritual connections with the dead and the living [11]. The Yoruba tradition highlights the cultural beliefs in the continuity of the human body after death [9]. All these belief systems have contributed to the scarcity of organ donation seen in many African countries [10], potentially impeding neuropathological research on CTE.

A multifaceted approach is essential to address these challenges. Proper awareness of the risk factors and symptoms of CTE is very important, as it will go a long way in sensitizing both people and health professionals to the disease and building the interest of prospective researchers in the area. Equally, CTE should also be included in the medical curriculum, especially in pathology, to educate medical students on the disease.

In addition, the government should provide funding opportunities to conduct large-scale studies. Similarly, case reports should be performed on former athletes who show symptoms consistent with CTE, and researchers should also engage with communities and religious leaders to sensitize them to the myths surrounding organ donation in general. This would dispel the myths and encourage popular participation in brain donation. Lastly, international collaboration on CTE should be encouraged as it facilitates skill and knowledge transfer, helping African researchers build their expertise.

In conclusion, action is urgently needed to prevent the potential Chronic Traumatic Encephalopathy (CTE) crisis in Africa. Global trends in CTE research have highlighted the mandates of early intervention, which could reduce additional strain on future healthcare and improve long-term outcomes for the at-risk population (athletes) in Africa.

## Data Availability

Not applicable.
